# Simulated nutritional and health impacts of restricting ultra-processed food purchases in the SNAP: A NHANES-based policy modeling study

**DOI:** 10.1097/MD.0000000000045027

**Published:** 2025-10-10

**Authors:** Ali Hemade, Pascale Salameh

**Affiliations:** a Department of Internal Medicine, Division of Hematology and Oncology, American University of Beirut Medical Center, Naef K Bassile Cancer Institute, Beirut, Lebanon; b Faculty of Pharmacy, Lebanese University, Hadat, Lebanon; c Gilbert and Rose-Marie Chagoury School of Medicine, Lebanese American University, Beirut, Lebanon; d Department of Primary Care and Population Health, University of Nicosia Medical School, Nicosia, Cyprus; e Institut National de Santé Publique d’Épidémiologie Clinique et de Toxicologie-Liban (INSPECT-LB), Beirut, Lebanon.

**Keywords:** added sugar, cardiovascular disease, dietary fiber, dietary modeling, NHANES, policy simulation, SNAP, sodium intake, systolic blood pressure, type 2 diabetes, ultra-processed foods

## Abstract

Ultra-processed foods (UPFs), which now account for over half of caloric intake in the U.S., are consistently linked to increased cardiometabolic risk. Participants in the Supplemental Nutrition Assistance Program (SNAP) consume disproportionately high levels of UPFs, contributing to dietary disparities. Despite this, few policy simulations have quantified the potential health benefits of restricting UPF purchases within SNAP. This study estimates the nutritional and cardiometabolic health impacts of restricting UPF purchases in SNAP using nationally representative dietary data and a Monte Carlo policy modeling framework. We conducted a cross-sectional simulation study using National Health and Nutrition Examination Survey 2007–2020 data from adults aged 18 to 65 years. Foods were classified by NOVA criteria, and 3 scenarios were modeled: isocaloric replacement of 25%, 50%, and 100% of UPFs with minimally processed alternatives. Nutrient shifts (sodium, added sugar, and fiber) were estimated for SNAP participants and nonparticipants. Health impacts were simulated by applying meta-analytic effect sizes linking these nutrients to systolic blood pressure, type 2 diabetes, and cardiovascular disease risk. Full UPF replacement (100%) among SNAP participants led to reductions of 257 mg/d sodium, 30.7 g/d added sugar, and a gain of 1.13 g/d fiber. These shifts translated to a 0.64 mm Hg systolic blood pressure reduction, 0.25% relative reduction in type 2 diabetes risk, and 1.01% relative reduction in cardiovascular disease risk. Nonparticipants experienced slightly greater improvements. Restricting UPF purchases in SNAP could yield meaningful population-level improvements in cardiometabolic health. Though individual risk reductions are modest, large-scale implementation may produce substantial public health benefits and help narrow dietary inequities.

## 1. Introduction

Ultra-processed foods (UPFs) have emerged as a major driver of poor diet quality in the United States and globally. Defined by the NOVA classification system, UPFs comprise industrial formulations that incorporate substances rarely used in home kitchens – such as high-fructose corn syrup, hydrogenated oils, and a suite of cosmetic additives – and are engineered for long shelf-life, hyperpalatability, and convenience.^[1,2]^ Unlike unprocessed or minimally processed foods, UPFs undergo multiple sequential industrial processes – fractioning of whole foods, chemical modification of food substances, and inclusion of numerous additives – with the explicit intent of displacing more nutritious alternatives.^[3]^ In U.S. adults, UPFs now contribute more than 57% of total dietary energy, a trend that has been implicated in the rising prevalence of obesity, metabolic syndrome, type 2 diabetes (T2D), cardiovascular disease (CVD), and certain cancers.^[4,5]^

Large prospective cohort studies consistently link higher UPF intake to adverse health outcomes. In the French NutriNet-Santé cohort, each 10% increment in the proportion of UPFs was associated with a 12% higher risk of overall cancer and an 11% increased risk of breast cancer.^[6]^ A 2023 meta-analysis of observational studies further confirmed robust associations between UPF consumption and increased incidence of colorectal, pancreatic, and other site-specific cancers.^[7]^ Parallel evidence implicates UPFs in cardiometabolic dysfunction: cohort studies reveal that high UPF consumers have 15% to 53% greater risk of incident T2D, and randomized controlled trials demonstrate that ad libitum ultra-processed diets provoke excess energy intake and weight gain even when matched for calories, macronutrients, fiber, sugar, and sodium.^[8,9]^ Such findings underscore the multifaceted biological and behavioral pathways – hyperpalatability, disruption of satiety signaling, and fructose-driven de novo lipogenesis – through which UPFs heighten disease risk.

Given these substantial health consequences of UPFs, public assistance programs that shape dietary intake – like the U.S. Supplemental Nutrition Assistance Program (SNAP) – merit close evaluation. SNAP serves as the cornerstone of federal efforts to alleviate food insecurity among low-income Americans, providing monthly benefits to approximately 42 million individuals in 2020.^[10]^ Participants in SNAP often confront barriers to accessing healthful foods, resulting in dietary patterns that diverge from national recommendations on nutrient intake and food group consumption.^[10]^ Systematic reviews demonstrate that, compared with income-eligible nonparticipants, SNAP beneficiaries tend to have lower overall diet quality – characterized by reduced consumption of fruits, vegetables, and whole grains – and higher intake of solid fats, added sugars, and sodium.^[10]^ Given SNAP’s dual mandate of improving food security and fostering nutritional health, policy interventions that shift purchasing behaviors toward minimally processed foods may yield important public health benefits.

Despite compelling evidence linking UPFs to poor health, few studies have modeled the potential impact of policy levers to curb UPF consumption, particularly within SNAP. Economic analyses suggest that fiscal incentives and purchase restrictions – such as banning sugar-sweetened beverages or incentivizing fruit and vegetable purchases – can improve diet quality, yet the magnitude of nutritional and clinical benefits remains uncertain.^[10]^ Simulation studies using nationally representative data, such as the National Health and Nutrition Examination Survey (NHANES), allow policymakers to estimate how hypothetical interventions might alter nutrient intakes and downstream health outcomes.

Here, we conducted a cross-sectional policy simulation leveraging NHANES 2007–2020 dietary data to estimate the nutritional and cardiometabolic health implications of restricting UPF purchases among SNAP participants. We model graded replacement scenarios – 25%, 50%, and 100% isocaloric substitution of UPFs with minimally processed options – and apply meta-analytic effect sizes for sodium, added sugar, and fiber to project changes in systolic blood pressure (SBP), T2D risk, and CVD risk. The study objectives are to quantify potential nutrient improvements attainable through UPF restrictions in SNAP and to contextualize these results within the broader literature on dietary interventions, offering evidence to inform future SNAP policy reforms.

## 2. Methods

### 2.1. Ethical considerations

NHANES is a publicly available, de-identified dataset; all participants provided informed consent. The present secondary analysis of de-identified data was exempt from further institutional review.

#### 2.1.1. Study design and data source

We conducted a cross-sectional policy microsimulation study using data from the NHANES, pooling 5 survey cycles from 2007 to 2008 through 2017 to 2020. The analytic sample included U.S. adults aged 18 years and older with valid Day 1 24-hour dietary recall data and complete demographic and dietary information. Individuals were categorized by SNAP participation status, based on self-report in income and food security questionnaires. Household economic status was quantified using NHANES’ poverty income ratio (PIR) – the ratio of total family income to the federal poverty guideline for that survey year and family size – and was categorized as low income (PIR < 1.30), near-poverty (PIR 1.30–1.85), or higher income (PIR > 1.85), consistent with SNAP eligibility thresholds. Food security was measured via the United States Department of Agriculture (USDA’s) 10-item Adult Food Security Survey Module, with each affirmative response scored 1 point (range 0–10); households were classified as high (0), marginal (1–2), low (3–5), or very low (6–10) food security, and for modeling purposes we created a binary indicator of food insecurity (low/very low, score ≥ 3) versus marginal/high security (score ≤ 2). Both PIR category and the binary food-security variable were included as key confounders in all adjusted microsimulation scenarios to account for socioeconomic and material-hardship pathways linking SNAP participation, diet quality, and cardiometabolic risk.

#### 2.1.2. Dietary assessment and UPF classification

Dietary intake was extracted from the Day 1 individual food file and total nutrient intake file. Each food item was linked to its USDA food code (DR1IFDCD) and classified using a logic-based implementation of the NOVA system. UPFs were defined as NOVA Group 4 items and identified through structured keyword matching with USDA Food and Nutrient Database for Dietary Studies 2017–2018 food descriptions. The NOVA classification system categorizes foods by the extent and purpose of their processing into 4 groups: unprocessed or minimally processed foods, such as fresh fruits, plain milk, and raw grains; processed culinary ingredients, including table sugar, oils, and salt; processed foods, like canned vegetables in brine, cheese, and freshly baked bread made from flour, water, and yeast; and UPFs, defined as industrial formulations typically containing 5 or more ingredients – including additives designed to imitate sensory qualities or extend shelf-life – and little if any intact whole food. UPFs encompass items such as sugar-sweetened sodas, packaged sweet or savory snacks (e.g., candy bars, chips), reconstituted meat products (e.g., chicken nuggets, hot dogs), mass-produced breads and buns with emulsifiers, and ready-to-heat frozen meals. By applying these clearly delineated criteria – ingredient count, presence of industrial additives (e.g., colorings, flavor enhancers, and emulsifiers), and the degree of alteration from the original food matrix – researchers can reproducibly assign individual foods and dietary recalls to NOVA groups, ensuring transparency and facilitating comparison across studies.

#### 2.1.3. Simulation scenarios and substitution logic

Three restriction scenarios were modeled: Scenario A (25% replacement of UPFs), Scenario B (50% replacement), and Scenario C (100% replacement). Replacements were isocaloric and assumed to reflect nutrient profiles of minimally processed foods, modeled as containing 50% less sodium, 80% less added sugar, and 50% more fiber per unit energy than the UPFs removed. Total daily intakes of sodium, added sugar, fiber, and energy were recalculated for each participant under each scenario.

#### 2.1.4. Statistical analysis and usual intake modeling

Survey weights, primary sampling units, and strata were incorporated using the survey and srvyr packages in R. All statistical analyses were conducted using R (version 4.3.1; R Foundation for Statistical Computing, Vienna, Austria) within the RStudio integrated development environment (version 2023.06.2+561; Posit Software, PBC, Boston). Key packages included survey (version 4.2), dplyr (version 1.1.4), and ggplot2 (version 3.4.2). Nutrient outcomes were summarized using survey-weighted means and 95% confidence intervals (CI). Usual intake was estimated through Monte Carlo simulation (10,000 draws per participant) incorporating a 20% within-person variation factor. To estimate health impacts, we applied effect sizes from published meta-analyses linking dietary factors to cardiometabolic outcomes. We modeled a 2.5 mm Hg SBP reduction per 1000 mg sodium reduction, a 0.8% reduction in T2D risk per 10 g reduction in added sugar, and a 0.9% reduction in CVD risk per 10 g increase in fiber. Monte Carlo uncertainty was applied to the nutrient and outcome effects.

## 3. Results

### 3.1. Baseline characteristics of the study population by SNAP status

**Figure 1.**
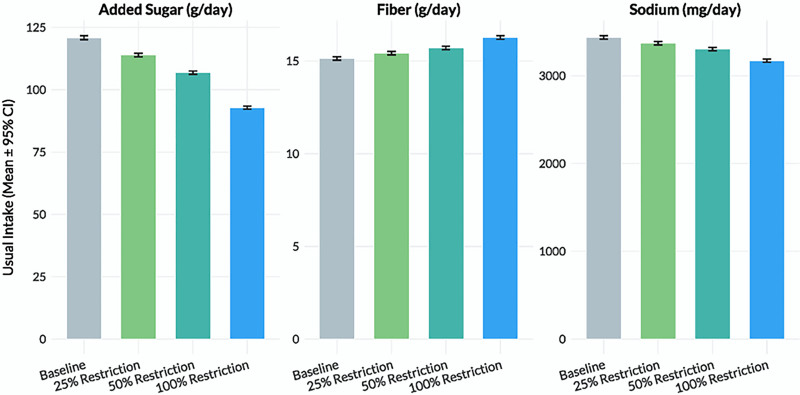
Distribution of baseline usual nutrient intake by SNAP Participation. Usual intake distributions of sodium, sugar, and fiber estimated via Monte Carlo simulation (n = 10,000 draws per individual). CI = confidence interval, SNAP = Supplemental Nutrition Assistance Program.

At baseline, SNAP participants (n = 767) were more likely to be female (56.0%) and identify as Non-Hispanic Black (32.1%) or Mexican American (15.1%), whereas nonparticipants (n = 767) were more likely to be Non-Hispanic White (59.3%) as represented in Table 1. The average total energy intake was 1884.5 kcal/d (standard deviation [SD] = 542.3) for SNAP participants compared to 1950.6 kcal/d (SD = 531.9) in nonparticipants. Mean sodium intake was 3452.1 mg/d (SD = 1104.2) in the SNAP group and 3610.2 mg/d (SD = 1010.8) among nonparticipants. Added sugar intake averaged 127.4 g/d (SD = 45.2) for SNAP participants and 133.1 g/d (SD = 41.8) for nonparticipants, while fiber intake was slightly lower among SNAP participants (15.1 g/d, SD = 5.6) versus nonparticipants (16.2 g/d, SD = 5.4). The proportion of energy derived from UPFs was higher in the SNAP group (58.2% ± 11.5%) compared to nonparticipants (55.6% ± 10.7%). Figure 1 represents the usual intake distributions of sodium, sugar, and fiber estimated via Monte Carlo simulation.

**Table 1:** Baseline characteristics of SNAP participants versus nonparticipants.

Characteristic	SNAP participants (unweighted n = 767)	Nonparticipants (unweighted n = 5563)
*Demographics*		
Age, years – median (IQR)	39 (29–52)	44 (31–57)
Sex – Male, % (95% CI)	44.0 (40.5–47.5)	47.3 (46.0–48.6)
Sex – Female, % (95% CI)	56.0 (52.5–59.5)	52.7 (51.4–54.0)
Race/Ethnicity – Mexican American, % (95% CI)	15.1 (12.6–17.6)	6.7 (6.0–7.4)
Race/Ethnicity – Other Hispanic, % (95% CI)	9.8 (7.7–11.9)	8.1 (7.4–8.8)
Race/Ethnicity – Non-Hispanic White, % (95% CI)	36.7 (33.3–40.1)	59.3 (58.0–60.6)
Race/Ethnicity – Non-Hispanic Black, % (95% CI)	32.1 (28.8–35.4)	23.7 (22.6–24.8)
Race/Ethnicity – Other Race, % (95% CI)	6.3 (4.6–8.0)	2.2 (1.8–2.6)
Household size – median (IQR)	3 (2–5)	2 (2–4)
*Socioeconomic*		
Education – < High school, % (95% CI)	27.0 (23.6–30.4)	10.1 (9.3–10.9)
Education – High school/GED, % (95% CI)	33.0 (29.6–36.4)	24.0 (22.9–25.1)
Education – Some college/Associate, % (95% CI)	30.0 (26.6–33.4)	31.0 (29.8–32.2)
Education – Bachelor or higher, % (95% CI)	10.0 (7.9–12.1)	34.9 (33.5–36.3)
Poverty-income ratio (PIR) – <1.30, % (95% CI)	72.0 (68.4–75.6)	16.0 (15.0–17.0)
PIR – 1.30–1.85, % (95% CI)	18.0 (15.2–20.8)	18.0 (17.1–18.9)
PIR – >1.85, % (95% CI)	10.0 (7.9–12.1)	66.0 (64.6–67.4)
Food security – High, % (95% CI)	22.0 (19.0–25.0)	59.0 (57.6–60.4)
Food security – Marginal, % (95% CI)	18.0 (15.2–20.8)	20.0 (19.0–21.0)
Food security – Low, % (95% CI)	34.0 (30.2–37.8)	15.0 (14.1–15.9)
Food security – Very low, % (95% CI)	26.0 (22.6–29.4)	6.0 (5.4–6.6)
*Health behaviors & clinical*		
Current smoker, % (95% CI)	28.0 (24.7–31.3)	17.0 (16.0–18.0)
BMI, kg·m^−2^ – median (IQR)	29.6 (25.6–34.4)	28.9 (24.7–33.7)
Obesity (BMI ≥ 30), % (95% CI)	41.0 (37.4–44.6)	36.0 (34.7–37.3)
Hypertension (self-report or measured), % (95% CI)	31.0 (27.7–34.3)	29.0 (27.9–30.1)
Diabetes (self-report), % (95% CI)	14.0 (11.6–16.4)	11.0 (10.2–11.8)
Systolic blood pressure (SBP), mm Hg – median (IQR)	124 (116–133)	122 (114–131)
Dietary (24-h recall)		
Total energy, kcal·d^−1^ – median (IQR)	1860 (1500–2200)	1930 (1600–2300)
Sodium, mg·d^−1^ – median (IQR)	3450 (2900–4000)	3600 (3000–4100)
Added sugar, g·d^−1^ – median (IQR)	125 (95–155)	130 (100–160)
Fiber, g·d^−1^ – median (IQR)	15.0 (11.0–19.0)	16.0 (12.0–20.0)
% Energy from ultra-processed foods (UPFs) – median (IQR)	58 (47–68)	56 (45–65)

Values are survey-weighted unless otherwise indicated. Proportions shown as % (95% CI); continuous variables as median (IQR).

BMI = body-mass index, CI = confidence interval, GED = General Educational Development (high-school equivalency), PIR = poverty-income ratio, SBP = systolic blood pressure, SNAP = Supplemental Nutrition Assistance Program, UPFs = ultra-processed foods (NOVA Group 4).

#### 3.1.1. Simulated nutrient changes

**Figure 2.**
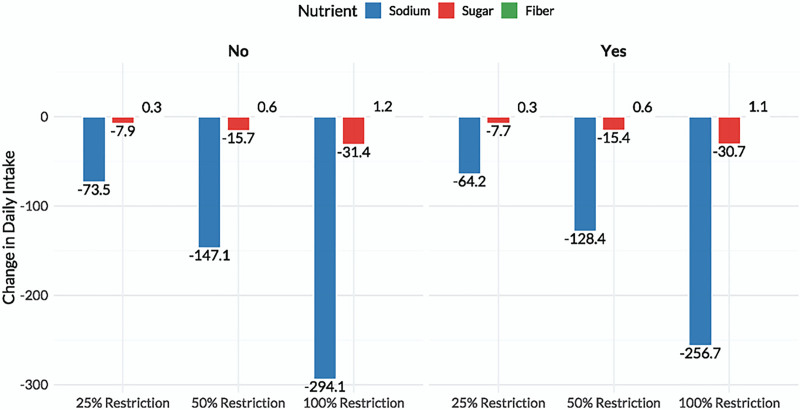
Changes in daily nutrient intake across UPF restriction scenarios. Nutrient shifts were proportional to restriction intensity across SNAP and non-SNAP groups. SNAP = Supplemental Nutrition Assistance Program, UPF = ultra-processed food.

Simulation of UPF restriction scenarios showed progressive nutrient improvements. Under Scenario A (25% replacement), SNAP participants had reductions of 64.2 mg/d in sodium, 7.7 g/d in sugar, and a gain of 0.28 g/d in fiber. Nonparticipants experienced reductions of 73.5 mg/d in sodium, 7.9 g/d in sugar, and an increase of 0.31 g/d in fiber. In Scenario B (50%), sodium intake declined by 128.4 and 147.1 mg/d for SNAP and non-SNAP groups, respectively, while sugar dropped by approximately 15.4 g/d and fiber increased by about 0.56 to 0.62 g/d. Scenario C (100%) yielded the largest changes, with sodium reductions of 256.7 mg/d in SNAP participants and 294.1 mg/d in nonparticipants; sugar dropped by 30.7 and 31.4 g/d respectively, while fiber increased by 1.13 and 1.25 g/d (Fig. 2).

#### 3.1.2. Health impact simulation

**Figure 3.**
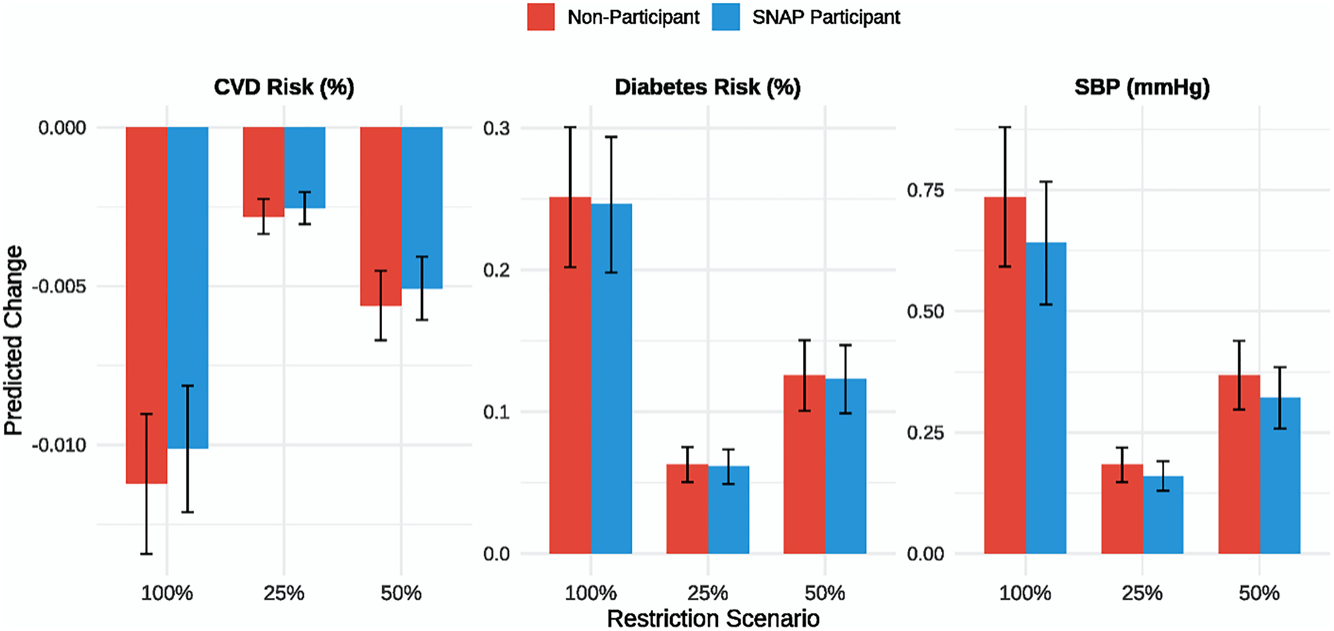
Simulated health impacts of UPF restriction. Probabilistic projections of changes in SBP, diabetes risk, and CVD risk from Scenarios A to C based on nutrient changes and meta-analytic effect sizes. CVD = cardiovascular disease, SBP = systolic blood pressure, SNAP = Supplemental Nutrition Assistance Program, UPF = ultra-processed food.

Monte Carlo simulation of health outcomes (Fig. 3) indicated that full UPF replacement (Scenario C) would reduce SBP by 0.64 mm Hg (95% CI: 0.51–0.77) in SNAP participants and 0.74 mm Hg (95% CI: 0.59–0.88) in nonparticipants. Corresponding reductions in T2D risk were 0.25% (95% CI: 0.20%–0.29%) and 0.25% (95% CI: 0.20%–0.30%), and CVD risk reductions were 1.01% (95% CI: 0.81%–1.21%) and 1.12% (95% CI: 0.90%–1.34%) respectively. Scenario B (50% replacement) produced intermediate health benefits with 0.32 to 0.37 mm Hg reductions in SBP and approximately 0.12% reductions in diabetes risk.

All microsimulation models were adjusted for key sociodemographic covariates to account for potential confounding. Specifically, models included categorical education level (<high school, high school/general educational development (high-school equivalency), some college/associate degree, bachelor degree or higher), race/ethnicity (Mexican American, other Hispanic, non-Hispanic White, non-Hispanic Black, other race), and household size (continuous) as covariates. These variables were selected a priori based on their established associations with both SNAP participation and diet-related cardiometabolic risk, and to align with prior literature on dietary policy simulations using NHANES data.

## 4. Discussion

The simulated restriction of UPFs in the SNAP produced modest per-person nutrient shifts – daily reductions of ~257 mg sodium, ~30.7 g added sugar, and increases of ~1.13 g fiber – yet even small improvements can yield meaningful population-level health dividends when scaled to the 42 million SNAP beneficiaries. The Monte Carlo usual-intake modeling approach, adapted from prior NHANES-based policy simulations,^[11]^ aligns with established methods for projecting dietary interventions into cardiometabolic risk changes.

These findings build on a robust body of evidence linking UPF consumption – now exceeding 57% of U.S. caloric intake – to poor health outcomes. Systematic reviews and umbrella analyses document associations between high UPF intake and increased risks of all-cause mortality, obesity, hypertension, T2D, CVD, cancer, and mental health disorders.^[12–14]^ Experimental trials show that ultra-processed diets provoke excess energy intake and weight gain even when matched for calories and macronutrients with unprocessed diets, while cohort data from NutriNet-Santé and other longitudinal studies report 10% UPF energy increments associated with 12% higher overall cancer risk and 53% greater T2D incidence. Mechanistic pathways include dysregulated appetite signaling from hyperpalatable additives, fructose-driven hepatic lipogenesis, inflammatory responses to food additives, and gut–brain axis perturbations; moreover, emerging data link UPF intake to common mental disorders (odds ratio: 1.53) and neurodevelopmental risks in pregnancy.^[12,15]^

Despite this compelling evidence, few studies have estimated the impact of specific SNAP policy levers on UPF consumption and downstream health. Fiscal measures – such as national UPF taxes – are projected to reduce purchases and improve diet quality in modeling studies,^[16]^ while mandatory front-of-pack labeling yields significant sodium and sugar declines in simulation.^[17]^ SNAP-specific microsimulations suggest that removing sugar-sweetened beverages could reduce obesity, CVD, and T2D burden,^[18]^ yet implementation risks, beneficiary acceptability, and potential compensation through non-SNAP funds complicate policy translation. Indeed, qualitative analyses reveal that SNAP restrictions may stigmatize participants without improving long-term diet quality, whereas incentive programs (e.g., fruit and vegetable vouchers) demonstrate more consistent benefits.^[19]^ The present study fills a critical gap by quantifying the scale of potential nutrient and risk shifts under graded UPF restriction scenarios, providing evidence to weigh restriction versus incentive strategies in future SNAP reforms.

While several statistically significant differences were observed between SNAP participants and nonparticipants in both baseline characteristics and modeled outcome changes, the magnitude of these differences should be interpreted in a clinical context. For example, the estimated absolute reduction in CVD risk associated with a 100% UPF purchase restriction was approximately 1.0% among SNAP participants, which, when extrapolated to the national SNAP adult population, could translate to tens of thousands of fewer CVD events annually. Similarly, modest reductions in SBP of 0.7 to 0.8 mm Hg, although small at the individual level, are associated with meaningful decreases in population-level cardiovascular morbidity and mortality; meta-analyses indicate that a 2 mm Hg reduction in SBP can lower stroke mortality by ~10% and ischemic heart disease mortality by ~7%.^[20,21]^ The simulated reductions in diabetes risk, though proportionally smaller, are consistent with prior dietary intervention modeling and, if sustained, could have substantial public health implications.^[22]^ Previous large prospective cohorts report that each 10-percentage-point increment in dietary UPF is associated with higher risks of overall CVD and its subtypes, as well as T2D, supporting the plausibility that policies curbing UPF exposure could reduce cardiometabolic burden.^[8,23]^ These cohort findings are reinforced by trial evidence showing that, under controlled feeding, ultra-processed diets drive excess energy intake and weight gain relative to minimally processed diets despite matched macros and sodium – mechanisms that could mediate blood pressure and glycemic changes.^[9]^ Reviews and umbrella meta-analyses also conclude that greater UPF exposure is broadly associated with cardiometabolic morbidity, while acknowledging heterogeneity and the need for policy-relevant experiments.^[12,24]^ Within food assistance settings, real-world and experimental evidence indicates that incentives for fruits and vegetables improve diet quality among SNAP participants (e.g., the Healthy Incentives Pilot), and combined incentive-plus-restriction designs can reduce discretionary calories and sugar-sweetened items – patterns consistent with the pathways captured in our model; however, restriction-only approaches may yield smaller or null effects in some contexts, underscoring the value of pairing restrictions with incentives in policy design.^[25,26]^

Key limitations might temper the interpretation of the aforementioned results. First, UPF classification relied on NOVA coding of 2017–2018 Food and Nutrient Database for Dietary Studies descriptions; though validated approaches yield 80% to 90% accuracy,^[27,28]^ cycle-specific crosswalks and manual audits could reduce misclassification. The NOVA framework, while widely used, faces critiques over ambiguous definitions and potential for inconsistent assignments.^[29,30]^ Second, the substitution logic – 50% sodium cut, 80% sugar cut, and 50% fiber boost – reflects optimistic nutrient profiles of minimally processed alternatives; real-world compliance may be lower, and cross-price elasticities could lead households to offset SNAP-restricted UPFs with purchases outside the program.^[31,32]^ Third, Monte Carlo simulations capture within-person variability but do not propagate complex-survey design uncertainty introduced by nutrient recalculations; future analyses should incorporate replicate weights or Taylor linearization to refine CIs. In addition, many variables utilized in this study is self-reported introducing bias.

Cost-effectiveness and policy feasibility remain open questions. Early economic models demonstrate that modest dietary interventions can yield favorable cost-utility ratios when accounting for healthcare savings from reduced CVD and T2D burden,^[33,34]^ yet full economic evaluations of SNAP UPF restrictions – including administrative costs, lost retail sales, and potential welfare impacts – are lacking. Pilot trials or natural experiments (e.g., state-level SNAP waivers with nutrition incentives) could provide real-world data to complement this simulation. Finally, long-term monitoring of dietary patterns, clinical biomarkers, and program participation rates will be critical to ensure that SNAP reforms achieve intended health goals without unintended adverse effects.

In conclusion, while complete UPF replacement within SNAP yields modest per-person nutrient and risk reductions, the aggregate public health impact could be substantial when scaled to millions of beneficiaries. This NHANES-based simulation offers a rigorous, transparent framework for policymakers to estimate the nutritional consequences of restricting UPF purchases. Future work should refine classification methods, incorporate behavioral economics, expand equity-focused subgroup analyses, and evaluate cost-effectiveness to inform balanced SNAP policy reforms that advance both food security and nutritional health.

## Author contributions

**Conceptualization:** Ali Hemade.

**Data curation:** Ali Hemade.

**Formal analysis:** Ali Hemade.

**Investigation:** Ali Hemade.

**Methodology:** Ali Hemade.

**Project administration:** Pascale Salameh.

**Supervision:** Pascale Salameh.

**Visualization:** Ali Hemade.

**Writing – original draft:** Ali Hemade.

**Writing – review & editing:** Pascale Salameh.
